# Supplementary material for: Bridging two insect flight modes in evolution, physiology and robophysics
Source: Nature. 2023 Oct 4;622(7984):767–74. doi: 10.1038/s41586-023-06606-3 (PMC10599994; doi:10.1038/s41586-023-06606-3)
Supplement: Supplementary file 1 — This file provides further information and extended discussions of the experiment, models, and results from the manuscript. [file 41586_2023_6606_MOESM1_ESM.pdf]

nature portfolio

https://doi.org/10.1038/s41586-023-06606-3

Supplementary information

Bridging two insect flight modes in evolution, physiology and robophysics

In the format provided by the
authors and unedited

Supplementary Discussion: Bridging two insect flight modes in evolution, physiology, and robophysics

Jeff Gau,[1,*] James Lynch,[2,*] Brett Aiello,[3,4,5,*] Ethan Wold,[4] Nick Gravish,[2,†] and Simon Sponberg[3,4,†]

[1]*Interdisciplinary Bioengineering Graduate Program, Georgia Institute of Technology, Atlanta, GA 30332, USA*
[2]*Department of Mechanical and Aerospace Engineering, University of California San Diego, San Diego, 92161, CA,USA*
[3]*School of Physics, Georgia Institute of Technology, Atlanta, GA 30332 USA*
[4]*School of Biological Sciences, Georgia Institute of Technology, Atlanta, GA 30332 USA*
[5]*Department of Biology, Seton Hill University, Greensburg, PA 15601 USA*

CONTENTS

A. Ancestral state reconstruction 2
1. Implementation 2
2. Comparison to other results 3
B. Delayed stretch activation model 4
1. Frequency-dependent power output of the delayed stretch activation feedback model 4
C. Dynamics of the asynchronous regime 6
1. Linear stability analysis of asynchronous dynamics 6
D. Simulation sensitivity analysis 8
1. Variation in K and F_s 9
2. Variation in κ 9
3. Comparison with and without body viscous damping 11

References 13

In this document we provide extended discussion we provide more context related to the analyses in the manuscript. In section A, we provide further information about the ancestral state reconstruction and additionally we provide the nearest alternative reconstructions as a sensitivity analysis. In section B, we provide a calculation of the work output from delayed stretch activation to compare with reported values in the literature. In section C, we analyze the asynchronous state using a linear systems analysis approach. Lastly, in section D, we provide a sensitivity analysis to model parameters.

[*] These authors contributed equally
[†] To whom correspondence should be addressed; E-mail: sponberg@gatech.edu, ngravish@ucsd.edu

A. Ancestral state reconstruction

1. Implementation

We used a phylogeny grounded in fossil records spanning all insect orders [1], which modifies the fossil calibration of the extensive insect phylogeny developed by Misof et al. [2]. For ancestral state reconstruction, we first assumed the most conservative model in terms of free parameters: an equal rates (ER) model of evolution. We used maximum likelihood estimation to estimate the posterior probability of ancestral states using the *Phytools* R Package [3]. These analyzes were performed in RStudio (v. 1.1.383) using R (v. 4.0.2). We report this reconstruction in the main text, but include a number of additional tests done with *corHMM* R package [4, 5].

We next tested if the reconstruction, especially if the pattern of single origin of asynchrony followed by multiple reversions to synchrony, was robust under other evolutionary models. In additional reconstructions, we allowed all the transition rates to differ (”all rates different”). Further, we alternatively coded ”unknown” and ”wingless” taxa as having their own unique character states (where wingless and unknown were character states) or, in other models, as being ambiguous with respect to synchronous and asynchronous fiber types and not having an assigned character state. Each of these additional reconstructions still produced the same transition patterns between character states at the order level, including the single ordinal original of asynchrony. Some small differences within orders with multiple transitions (e.g. Hemiptera) can occur, but more confident within-order resolution would likely require increased sampling that is currently unavailable for muscle ultrastructure data.

Adding more character states and allowing rates to be different can rapidly increase the number of free parameters in the rate transition matrix. We assess this with the Akaike information criterion corrected for sampling size (AICc). The most favored model treats ”unknown” and ”wingless” taxa as ambiguous (coded as ”?”) and has all rates differing (ARD vs ER AICc, 89.5 vs. 100.6). In this model Node 200 is reconstructed as the only origin of asynchrony 100% of the time. However, we are concerned that with 10 transitions across the phylogeny that the fitting of six rate parameters may still be overfit and fragile to additional sampling, so we report the more conservative single parameter, equal-rates model in the main paper. However, the single original transition from synchronous to asynchronous followed by multiple reversions and the status of Lepidoptera as secondarily synchronous fliers were consistent across all ancestral state reconstructions regardless of the specific model used or the specific coding of wingless and unknown taxa.

All the above models assume a single class of rates, that is homogenous rates of evolution over the entire phylogeny. However, especially across large phylogenies, the transition rates between character states may themselves vary across the phylogeny (e.g. one clade can evolve faster than another) [5]. These rate transitions can significantly affect ancestral state reconstructions and transition events, often improving models at least when sufficient data are available [4, 6]. To account for this possibility we also implement a hidden rates model that includes transitions between 2 or more crates of evolution across the phylogeny using the multiple rate classes models in the *corHMM* R package. Given that the introduction of a second hidden rate transition at least doubles the number of transition rates that must be estimated by the model, we suspected that these more complex models would overfit the data because there were only 10 transitions across the whole phylogeny. This was indeed the case, as the resulting AICc values were higher with hidden rates included. The AICc for the ARD model for all character states coded was 242.2 with two classes and only 183.8 with one class. With ambiguous coding for ”unknown” and ”wingless” taxa, the AICc was 100.4 for two rate classes compared to 89.5 for one class. An ER model with multiple rate classes did have a more favorable AICc to the one class ER model (AICc 94.3 vs. 100.6 respectively), but if the model is going to be made more complex, the ARD model with one

class of rates is still favored (AIC 89.5).

The overfit reconstructions with multiple rate classes did produce more variable transitions, with many patterns possible across separate stochastic character reconstructions. Single origins of asynchrony were still common, although not the majority specifically at Node 200. The majority of reconstructions had an asynchronous ancestor at a node basal to lepidopterans. Reconstructions might improve with more sampling but many orders are likely exclusively synchronous or asynchronous, and there are unlikely to be too many more transitions. We do suspect that evolutionary rates may differ over the tree, especially in Hemiptera where multiple transitions occur. This would be consistent with different orders typically occupying parts of parameter space near or far away from the bridge as seen in simulations and robophysical experiments (Fig. 3). We just cannot support a more accurate reconstruction with this assumption given the limitations in the data available. Regardless the phylogenetic evidence that at least some clades can transition and potentially share asynchronous and synchronous properties is independently supported by our subsequent physiology experiments on lepidopteran flight muscle (Fig. 2).

2. Comparison to other results

Previous attempts to infer the evolutionary history of muscle type across all insects have equivocally concluded that asynchronous flight muscle has evolved independently multiple times [7]. However, at the time of these previous studies, a well-resolved insect phylogeny was not available and the position of key groups has since been further resolved. In light of this, [7] acknowledges that other evolutionary scenarios are possible and points out that in their own work, the assignment of asynchronous muscle to key equivocal ancestral branches would result in a single origin of asynchronous flight muscle with several reversions back to secondarily synchronous flight muscle [7]. Our present study does indeed find that the equivocal ancestral branches noted by [7] to be asynchronous flight muscle, which does result in a single origin of asynchronous flight muscle, supporting one of the alternative hypotheses by [7]. We then further support these findings through the identification of key physiological features associated with asynchronous muscle (delayed stretch activation) in the secondarily synchronous hawkmoth flight muscle (see main text). Finally, a single origin of asynchronous flight muscle does not preclude further specialization of asynchronous muscle and differences in the physiology and ultrastructure of asynchronous flight muscle between insect orders. It is highly likely that asynchronous flight muscle has indeed continued to evolve since its single origin and further specializations within given orders are expected based on the functional demands of the evolution of new behaviors and ecological niches between orders.

B. Delayed stretch activation model

1. *Frequency-dependent power output of the delayed stretch activation feedback model*

The work and power output from muscle is often characterized through a "work loop" experiment in which muscle is subjected to a sinusoidal length change and the force is measured [8, 9]. Cycle-averaged work and power output are computed by integrating the forces vs position over a cycle. This technique can be used both in for synchronous muscle contractions where the force is primarily due to neurogenic release of calcium, or stretch-activated asynchronous muscle contractions [10]. A characteristic phenomenon in asynchronous muscle is that the work and power output are maximized at an oscillatory frequency between zero and an upper limit maximum.

Here, we compare the work and power output predictions from our convolution model of delayed stretch activation to assess the model's efficacy in recapitulating physiological properties. Assuming a sinusoidal length input, we can analytically calculate the power output of a delayed stretch activation feedback model.

As defined in the main text, we defined asynchronous forcing (F_{async}) as

$$F_{async}(\dot{\varepsilon}, t) = \mu F_a(-g * \dot{\varepsilon})(t) = -\mu F_a \int_0^t \dot{\varepsilon}(\tau) g(t-\tau) d\tau, \tag{1}$$

The nondimensional kernel $g(t)$ is defined in the methods and we repeat it below

$$g(t) = \frac{1}{g_0}\left(-e^{-r_3 t} + e^{-r_4 t}\right) \tag{2}$$

We evaluate the convolution (Eq. 1) assuming a sinusoidal strain rate ($\dot{\varepsilon}(t) = \varepsilon_0 \omega \cos(\omega t)$), ε_0 is the strain oscillation amplitude, and ω is the imposed oscillatory frequency. This yields the following equation for the time dependence of the delayed stretch activation force

$$\begin{aligned} F_{async} = &-\mu F_a \frac{\varepsilon_0 \omega^2}{g_o}\left[\frac{1}{\omega^2 + \kappa^2 r_3^2} - \frac{1}{\omega^2 + r_3^2}\right]\sin(\omega t) \\ &-\mu F_a \frac{\varepsilon_0 \omega r_3}{g_o}\left[\frac{\kappa}{\omega^2 + \kappa^2 r_3^2} - \frac{1}{\omega^2 + r_3^2}\right]\cos(\omega t) \\ &+\mu F_a \frac{\varepsilon_0 \omega r_3}{g_o}\left[\frac{\kappa e^{-\kappa r_3 t}}{\omega^2 + \kappa^2 r_3^2} - \frac{e^{-r_3 t}}{\omega^2 + r_3^2}\right] \end{aligned} \tag{3}$$

where $\kappa = r_4/r_3$. We are interested in the steady-state periodic response of the muscle after transients have died away, and so we can neglect the exponential decay in the third term. To calculate work (W) we integrate the instantaneous power (force times velocity, $L\dot{\varepsilon}$) over a cycle

$$W = \int_0^{2\pi/\omega} (F_{async}) \times (L\dot{\varepsilon})\, dt \tag{4}$$

$$= \mu F_a L \frac{\varepsilon_0^2 \omega^2 r_3}{g_o}\left[\frac{1}{\omega^2 + r_3^2} - \frac{\kappa}{\omega^2 + \kappa^2 r_3^2}\right]\int_0^{2\pi/\omega} \cos^2(\omega t) dt \tag{5}$$

$$= \mu F_a L \frac{\varepsilon_0^2 \omega^2 r_3}{g_o}\left[\frac{\kappa}{\omega^2 + \kappa^2 r_3^2} - \frac{1}{\omega^2 + r_3^2}\right]\left(\frac{\pi}{\omega}\right) \tag{6}$$

$$= \mu F_a L \frac{\pi \varepsilon_0^2 \omega r_3}{g_0}\frac{\left(\omega^2 - \kappa r_3^2\right)(1-\kappa)}{\left(r_3^2 + \omega^2\right)\left(\kappa^2 r_3^2 + \omega^2\right)} \tag{7}$$

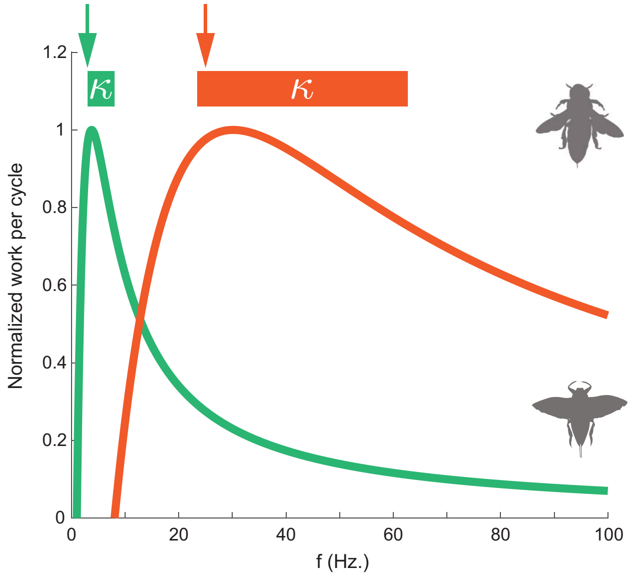

FIG. SI1. **Predicted work output per cycle from delayed stretch activation model**. The two curves show predictions from Equation 9 for *Lethocerus indicus* (green; $r_3 = 20$ s^{-1}) and *Vespula vulgaris* (orange; $r_3 = 160$ s^{-1}). Values of r_3 are from [11]. A value of $\kappa = 0.1$ recapitulates the frequency of maximum work output compared with experiment (arrows above plot[11]). Since κ is not directly known we also include the full frequency range of work output as horizontal bars for the feasible range of $\kappa = [0, 1]$.

The work output is still dependent on our normalization parameter (g_o). Because we normalized the convolution by the area under the curve in our simulations and experiments, we solved for g_o as

$$g_o = \int_0^\infty g(t)dt = \frac{(1-\kappa)}{r_3\kappa} \tag{8}$$

Normalizing Eq. 7 by g_o gives the final work output yields

$$W = \mu F_a L \frac{\pi \varepsilon_0^2 \kappa \omega r_3^2 \left(\omega^2 - \kappa r_3^2\right)}{\left(r_3^2 + \omega^2\right)\left(\kappa^2 r_3^2 + \omega^2\right)} \tag{9}$$

From this equation, we conclude that work output is positive if $\omega > \sqrt{\kappa} r_3$. Intuitively this makes sense: if the muscle is stretched slowly (small ω) then it will develop a delayed stretch activation force that resists the stretching, thus causing a negative work output (dissipating energy). However, if the muscle is stretched fast enough the delayed rise in tension will occur during the contraction cycle which follows the stretch. In this case, the delayed stretch activation force will pull while the muscle is shortening and will thus do positive work to power wingbeats.

To assess the efficacy of this model and assumptions made in the main text, we used r_3 values from [11] for *Vespula vulgaris* and *Lethocerus indicus*. Note that *Vespula vulgaris*, the common wasp, is sometimes referred to as *Vespa vulgaris*. For both species, we calculated cycle-averaged work output over a range of frequencies. We find that our model recapitulates the basic observed phenomenon of delayed stretch activation, work output is maximized at intermediate oscillatory frequency (Fig. SI1). Furthermore, the predictions from Equation 9 for the frequency where maximum work occurs agrees well with measurements from Molloy (arrows in Fig. SI1 are measured frequencies of maximum work from [11]). Therefore, our dSA feedback model captures the essential frequency dependent properties of asynchronous muscle. This also gives a framework for comparing the delayed stretch activation parameters from the stretch-and-hold experiments to the power output possible in cyclical wingbeats.

C. Dynamics of the asynchronous regime

1. *Linear stability analysis of asynchronous dynamics*

In this section, we will transform the convolution representation of delayed stretch activation into an ordinary differential equation. By using a differential equation as the mathematical representation of delayed stretch activation we can apply analysis tools from nonlinear dynamics to explore the basic properties of asynchronous spring-wing dynamics. The delayed stretch activation force, F_{async} is naturally represented as a convolution of a dSA kernel, $g(t)$, with the strain rate of the muscle, $\dot{\varepsilon}$.

$$F_{async}(\dot{\varepsilon}, t) = \mu F_a(-g * \dot{\varepsilon})(t) \tag{10}$$

We say "naturally", because the kernel $g(t)$ can be directly observed from stretch and hold experiments on insect muscle. The kernel is typically fit with a multi-rate and exponential equation that as described in the methods and reduced to a normalized two parameter model in Eq.2 in the previous section. The kernel is normalized by the area under the curve, $\frac{1}{g_0} = \frac{\kappa r_3}{1-\kappa}$ from Eq. 8 and we allow for a scaling parameter μ. To convert this convolution expression to a differential equation we can work in the Laplace domain. The Laplace transform of a convolution yields a straightforward multiplication rather than an integration, i.e. $F_{async}(s) = \mu F_a G(s)\dot{\mathcal{E}}(s)$, where s is the Laplace variable. Taking the Laplace transform of the delayed stretch activation kernel, $g(t)$, yields the transfer function $G(s)$ which transforms the velocity feedback input to the delayed stretch activation response:

$$\begin{aligned}\mathcal{L}(g(t)) = G(s) &= \frac{1}{g_0}\left(\frac{-r_3(1-\kappa)}{s^2 + r_3(1+\kappa)s + \kappa r_3^2}\right) \\ &= \frac{-\kappa r_3^2}{s^2 + r_3(1+\kappa)s + \kappa r_3^2} &(11)\\ &= \frac{-\alpha_3}{s^2 + \alpha_2 s + \alpha_3} &(12)\end{aligned}$$

where we have used the following definitions: $\alpha_2 = r_3(1+\kappa)$, $\alpha_3 = \kappa r_3^2$. Once we have the delayed stretch activation dynamics represented in the Laplace domain, we can express the dynamics of asynchronous actuation as an ODE. In the Laplace domain, the force output is the product of the transfer function and the velocity input:

$$F_{async}(s) = \mu F_a \left[\frac{-\alpha_3}{s^2 + \alpha_2 s + \alpha_3}\right] \dot{\mathcal{E}}(s) \tag{13}$$

We can distribute the denominator of the transfer function and take the inverse Laplace transform:

$$s^2 F_{async}(s) + \alpha_2 s F_{async}(s) + \alpha_3 F_{async}(s) = -\mu F_a \alpha_3 \dot{\mathcal{E}}(s) \tag{14}$$

$$\mathcal{L}^{-1} \Rightarrow \ddot{F}_{async} + \alpha_2 \dot{F}_{async} + \alpha_3 F_{async} = -\mu F_a \alpha_3 \dot{\varepsilon} \tag{15}$$

Equation 15 represents a linear ordinary differential equation form of the delayed stretch activation phenomena. This is identical to the convolution formulation and yields identical results. However, the ODE form of these equations leads to a straightforward analysis of the dynamics when delayed stretch activation muscle is coupled to the body mechanics.

We now examine the case when only asynchronous actuation occurs, $K_r = 0$. In this case the muscle force, $F_m = F_{async}(\dot{\varepsilon}, t)$. The delayed stretch activation force dynamics and the spring wing dynamics yield a pair of coupled, second order differential equations.

$$I\ddot{\phi} + \Gamma|\dot{\phi}|\dot{\phi} + \frac{k_l \phi}{T^2} = \frac{F_{async}}{T} \tag{16}$$

$$\ddot{F}_{async} + \alpha_2 \dot{F}_{async} + \alpha_3 F_{async} = -\mu F_a \alpha_3 \dot{\phi} \tag{17}$$

This set of coupled equations now allows to study the basic dynamics of emergent oscillatory wingbeats in asynchronous flight. We begin by transforming this into a coupled set of first order differential equations with the following transformations $\boldsymbol{\sigma} = [\theta, \dot{\theta}, f_{dSA}, \dot{f}_{dSA}]$ such that

$$\begin{array}{rcl} \dot{\sigma}_1 &=& \sigma_2 \\ \dot{\sigma}_2 &=& -\frac{k}{T^2 I}\sigma_1 - \frac{\Gamma}{I}|\sigma_2|\sigma_2 + \frac{1}{TI}\sigma_3 \\ \dot{\sigma}_3 &=& \sigma_4 \\ \dot{\sigma}_4 &=& -\mu F_a \alpha_3 \sigma_2 - \alpha_3 \sigma_3 - \alpha_2 \sigma_4 \end{array} \tag{18}$$

We observe that the only fixed point of this system is the state, $\boldsymbol{\sigma} = [0, 0, 0, 0]$ at the origin of the phase space, which corresponds to the stationary, unmoving state of system where no wingbeats occur. To determine how wingbeats emerge in the asynchronous state we first linearize the system around the origin and determine its stability. If the origin is stable, then small perturbations will die down to rest at the fixed point and no wingbeats will occur (note, linear stability does not guarantee this globally over the phase space). If the origin is unstable then small perturbations will grow and eventually be counterbalanced by the nonlinear damping from aerodynamics. Linear stability analysis determines when perturbations grow and the emergent oscillatory properties of the system.

We linearize the system by neglecting only the aerodynamic damping which scales as the square of small perturbation amplitudes. This leads to the following state matrix that describes the linear dynamics

$$\dot{\boldsymbol{\sigma}} = \begin{bmatrix} 0 & 1 & 0 & 0 \\ -\frac{k}{T^2 I} & 0 & \frac{1}{TI} & 0 \\ 0 & 0 & 0 & 1 \\ 0 & -\mu F_a \alpha_3 & -\alpha_3 & -\alpha_2 \end{bmatrix} \boldsymbol{\sigma} \tag{19}$$

Stability and oscillatory frequency are determined by the eigenvalues of this equation. We first determine the boundary between a stable (i.e. no oscillations) and unstable (i.e. oscillations) origin by evaluating the characteristic equation

$$\lambda^4 + \alpha_2 \lambda^3 + \left(\frac{k}{T^2 I} + \alpha_3\right)\lambda^2 + \left(\frac{k}{T^2 I}\alpha_2 + \frac{\mu F_a}{TI}\alpha_1\right)\lambda + \frac{k}{T^2 I}\alpha_3 = 0 \tag{20}$$

Examination of the real and imaginary components of the eigenvalues reveal that the instability that leads to asynchronous oscillations occurs through a Hopf bifurcation in which a pair of complex conjugate eigenvalues for the linearized system go unstable. The boundary between this (in)stability is determined when the eigenvalues have no real component, $\lambda = i\omega$ which results in two equations

$$\begin{array}{ll} \textbf{Real}: & \omega^4 - (\frac{k}{T^2 I} + \alpha_3)\omega^2 + \frac{k}{T^2 I}\alpha_3 = 0 \\ \textbf{Imag}: & \frac{k}{T^2 I}\alpha_2 + \frac{\mu F_a}{TI}\alpha_3 - \alpha_2 \omega^2 = 0 \end{array} \tag{21}$$

The first equation can be solved for ω^2 such that $\omega^2 = \alpha_3 \quad (\oplus)$ and $\omega^2 = \frac{k}{T^2 I} \quad (\ominus)$ and substitution into the second equation yields the relationships

$$\ominus \rightarrow \mu F_a = 0 \tag{22}$$

$$\oplus \rightarrow \frac{k}{T^2 I}\alpha_2 + \frac{\mu F_a}{TI}\alpha_3 - \alpha_2\alpha_3 = 0 \tag{23}$$

The first equation indicates that a change in stability may occur as the sign of the delayed stretch activation force changes. However, in a muscle-driven system, $F_a > 0$, indicating tension force only. Thus, we focus on the second equation. We can substitute in the definitions of $\alpha_{2,3}$ and define the natural frequency of the system (in rad s^{-1}) as $\omega_n^2 = \frac{k}{T^2 I}$.

$$\kappa(1+\kappa)r_3^2 - \kappa\frac{\mu F_a}{TI}r_3 - (1+\kappa)\omega_n^2 = 0 \tag{24}$$

This can be solved for r_3 through the quadratic equation. Further, we ignore the negative valued solution, which would produce an $r_3 < 0$ root and is unphysical. Thus, the onset of instability is determined by the following relationship

$$r_3 = \frac{\kappa\frac{\mu F_a}{TI} + \sqrt{\left(\kappa\frac{\mu F_a}{TI}\right)^2 + 4\kappa(1+\kappa)^2\omega_n^2}}{2\kappa(1+\kappa)} \tag{25}$$

Lastly, we subsitute the relationship for t_0 (for hawkmoth parameters) into this equation yielding the final relationship

$$t_0 = 1.258\frac{2\kappa(1+\kappa)}{\kappa\frac{\mu F_a}{TI} \pm \sqrt{\left(\kappa\frac{\mu F_a}{TI}\right)^2 + 4\kappa(1+\kappa)^2\omega_n^2}} \tag{26}$$

In Figure SI2 we demonstrate the validity of the linear analysis. We plot the amplitude and frequency of the asynchronous simulation ($K_r = 0$) and we also plot the real and imaginary components of the largest eigenvalue. We see that the imaginary component of the dominant eigenvalue matches the emergent frequency from the full nonlinear system extremely well. Furthermore, the onset of instability agrees well with the prediction in Equation 26. For small t_0, the muscle response is very "fast" and thus will generate tension force during the stretch component of a cyclic displacement. This resistance to stretch will dissipate energy and will not produce oscillations. However, as the time-to-peak of the force response increases, and the muscle becomes "slower" in response, then a cyclic displacement will produce a time-lagged rise in force that will pull in tension during the contraction phase of the cyclic displacement and will produce work over a cycle that drives emergent oscillations.

D. Simulation sensitivity analysis

In this section, we provide additional simulation results to demonstrate that the fundamental results are not sensitive to system parameters. We first vary the stiffness and synchronous forcing parameters to account for potential increased stiffness from muscle elasticity. In the second section, we vary the asynchronous parameter κ which determines the relative rates of delayed tension rise (r_3) and fall (r_3).

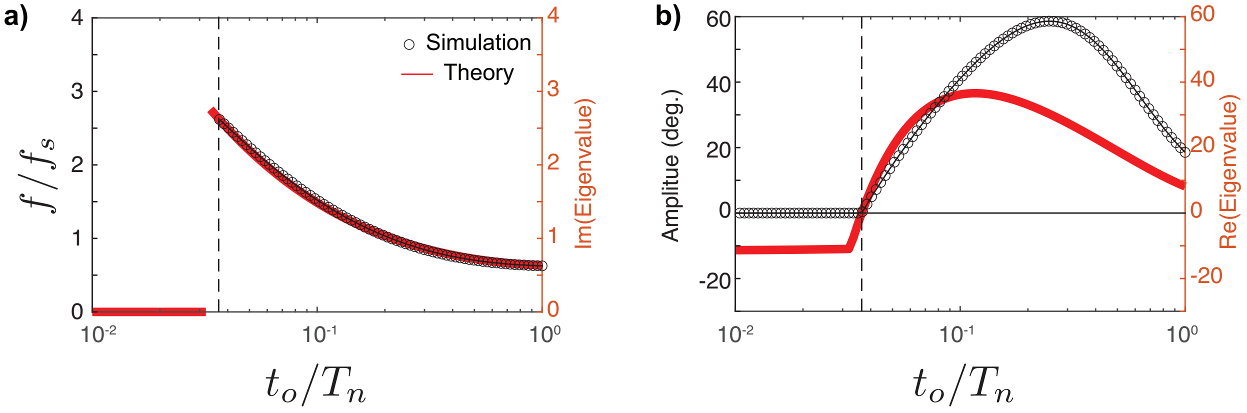

FIG. SI2. **Comparison of the asynchronous dynamics from the linearized and nonlinear solutions**. The vertical dashed line on both plots corresponds to the prediction from Equation 26 for the onset of emergent oscillations from the linear stability analysis. a) Left axis and circles correspond to the emergent wingbeat frequency normalized by synchronous frequency versus the normalized delayed stretch activation time to peak, t_o/T_n. The right axis and red line correspond to the imaginary component of the largest eigenvalue in the linearized system. b) Left axis and circles correspond to emergent wingbeat amplitude versus the normalized delayed stretch activation time to peak, t_o/T_n. The right axis and red line correspond to the real component of the largest real eigenvalue. When the eigenvalue with the largest real component becomes positive, stable limit-cycle wingbeats occur.

1. Variation in K and F_s

In our main results we do not consider additional elasticity contributions that may come from the muscle. We have previously determined that *passive* muscle does not contribute to the elasticity of the thorax system [12]. However, it is unknown if *active* muscle during contractions may induce an additional elasticity to the thorax, although flight muscle does have the capacity to store and return energy [13]. To estimate this we followed a procedure from recent work [14] where we estimated the contribution of the antagonistic pair of flight muscles as twice the active stiffness of the main downstroke [13] muscles (DLM) found by fitting a stiffness to work loop data [15]. We then tuned the synchronous force, F_s, so that the simulation generated realistic wingbeat amplitudes of 117° under synchronous forcing. In the simulation that estimates active muscle elasticity, all parameters are the same except, $k = 4078$ N m^{-1} and $F_s = 2250$ N.

Figure SI3 demonstrates the comparison with and without active muscle stiffness. The fundamental results are the same in both simulations, there exists a bridge in parameter space between the purely asynchronous and synchronous modes where high-power and steady wingbeats are generated. At the boundaries of this bridge, the wingbeats show large fluctuations in peak-to-peak amplitude. These results indicate that the qualitative behavior of synchronous plus asynchronous actuation is preserved even under conservative assumptions about body and muscle mechanics.

2. Variation in κ

In our simulations and experiment, we varied a single parameter related to delayed stretch activation, the time to peak, t_0. However, there is a second parameter (κ) that governs the delayed stretch activation force response in our model. The parameter κ determines the ratio between the tension rise rate constant (r_3) and the tension relaxation rate constant (r_4) such that $r_3 = \kappa r_4$. For a delayed stretch activation response the tension rise rate constant must be larger than the

relaxation rate constant, and so κ is bounded $\kappa \in [0, 1]$. In hawkmoth experiments, we found $\kappa = 0.62$, which is what was used in the main paper simulations. Here we also present simulations with $\kappa = 0.1$ and $\kappa = 0.9$ to explore how variation in this parameter influences the observed phenomenon.

Figure SI4 illustrates the comparison of emergent frequency, power, and amplitude fluctuations for three different values of κ. For a low $\kappa = 0.1$ we observe a wider regime of asynchronous oscillations across t_0/T_n, and as κ is increased this regime of asynchrony becomes smaller and the value of t_0/T_n for the onset of asynchronous oscillations increases. However, across all three values of κ we observe the same qualitative features–a bridge of high-power and low amplitude fluctuations wingbeats exists between the synchronous and asynchronous modes. These simulations indicate that our qualitative results are not sensitive to κ.

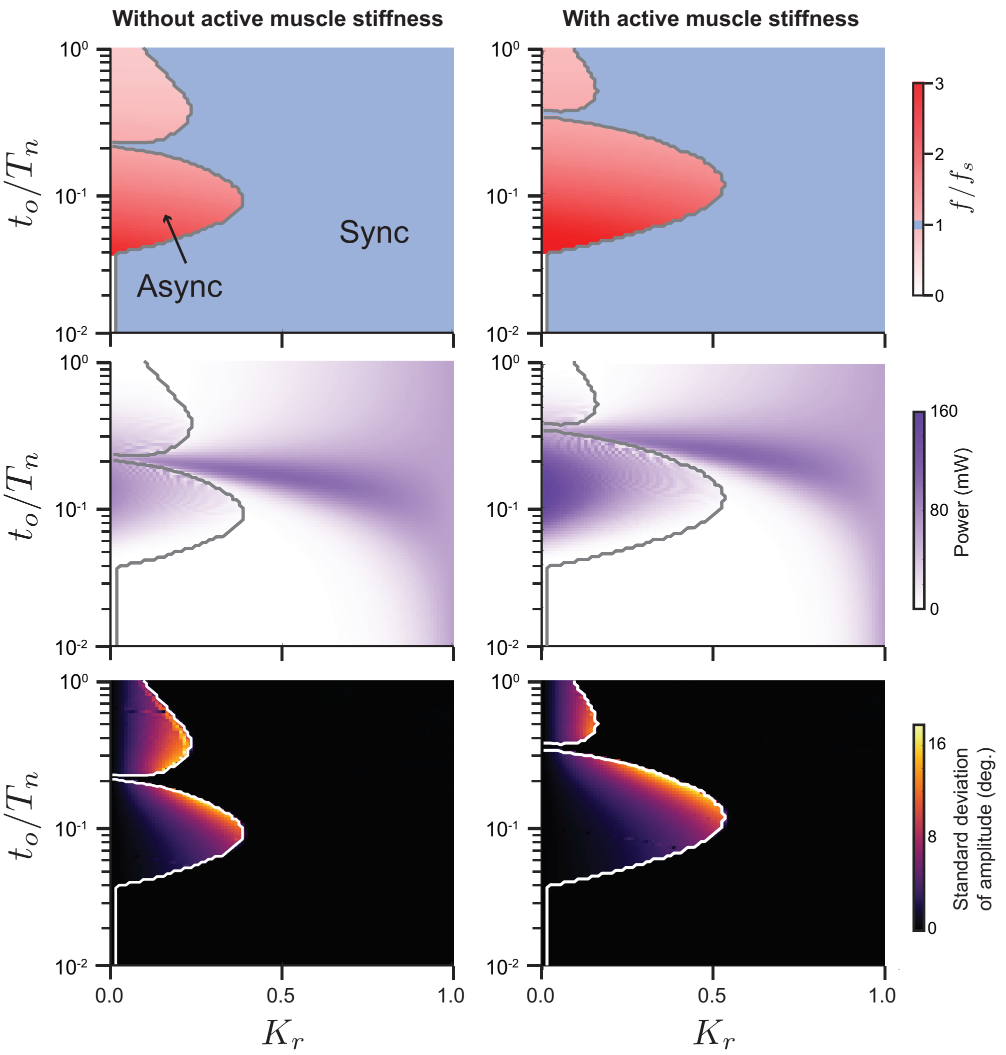

FIG. SI3. **Comparison of simulation results with and without active muscle elasticity.** Top row shows emergent frequency and bottom row shows the standard deviation of peak-to-peak amplitude. White lines designate boundary between asynchronous and synchronous modes. Both simulations display the same fundamental phenomena: 1) asynchronous oscillations emerge as the normalized time-to-peak of the delayed stretch activation response $(t_0/T)n)$ increases, and 2) a bridge of smooth sinusoidal wingbeats exists between the asynchronous and synchronous modes of actuation.

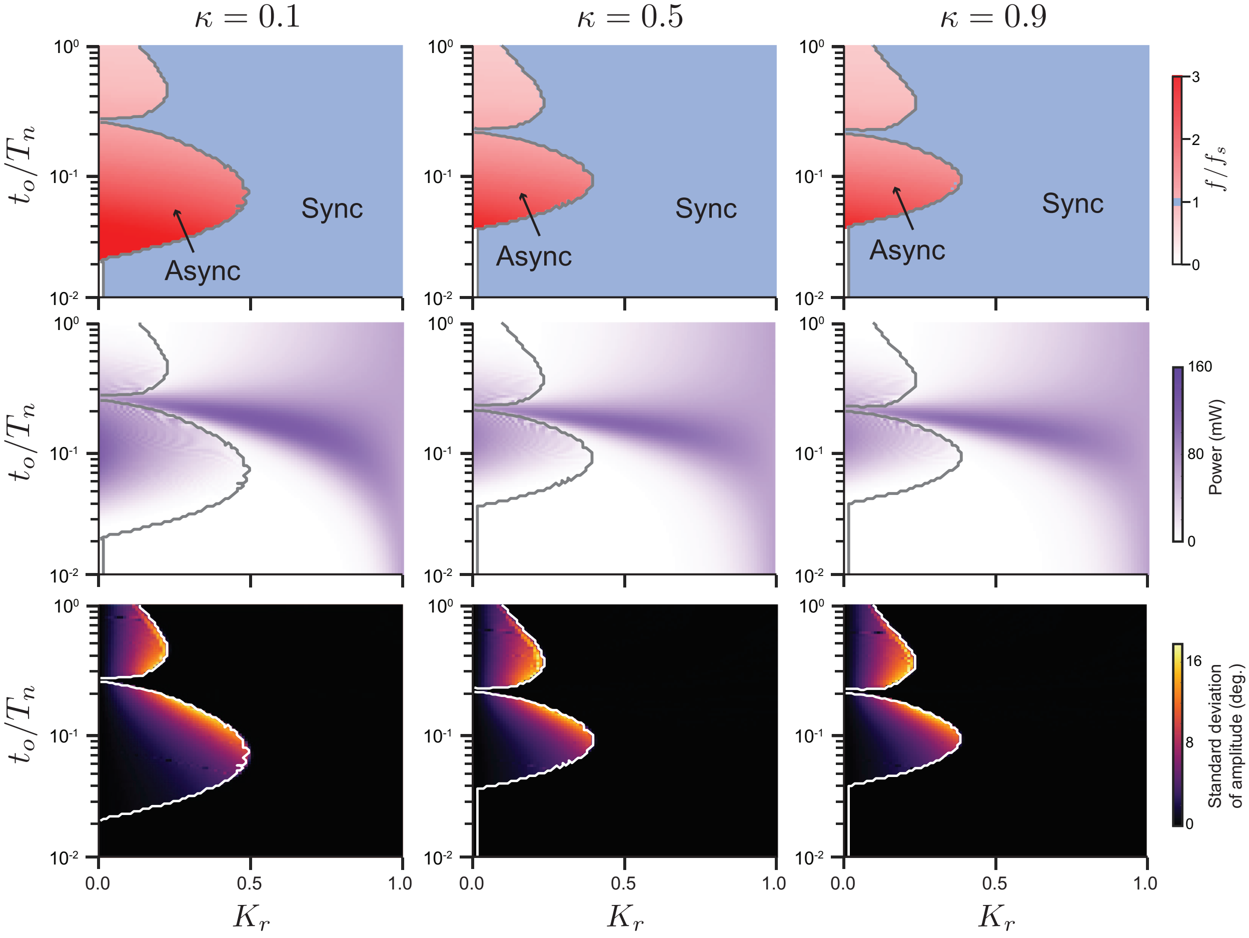

FIG. SI4. **Comparison of simulation results with different ratio of rate constants r_3 and r_4 ($r_3 = \kappa r_4$).** Top row shows emergent frequency, middle row shows power, and bottom row shows the standard deviation of peak-to-peak amplitude. White lines designate boundaries between asynchronous and synchronous modes. All simulations display the same fundamental phenomena: 1) asynchronous oscillations emerge as the normalized time-to-peak of the delayed stretch activation response (t_0/T_n) increases, and 2) a bridge of smooth sinusoidal wingbeats exists between the asynchronous and synchronous modes of actuation.

3. Comparison with and without body viscous damping

In the robophysical experiments, we observed that at the upper range of the asynchronous regime the system could not produce oscillations. We speculated this was due to frictional dissipation in the system that delayed stretch activation was not able to overcome. In insects, muscle and thorax deformation can induce energy loss, whereas in robots Coulomb friction and viscosity may induce energy. Thus, to examine how energy dissipation influences our results we included a viscous damping term in the body mechanics equation and performed new simulations

$$\frac{F_m}{T} = I\ddot{\phi} + \Gamma|\dot{\phi}|\dot{\phi} + \frac{b}{T^2}\dot{\phi} + \frac{k}{T^2}\phi. \tag{27}$$

We characterize the amount of viscous body dissipation through the standard damping ratio for harmonic oscillators

$$\xi = \frac{b}{2T\sqrt{Ik}}. \tag{28}$$

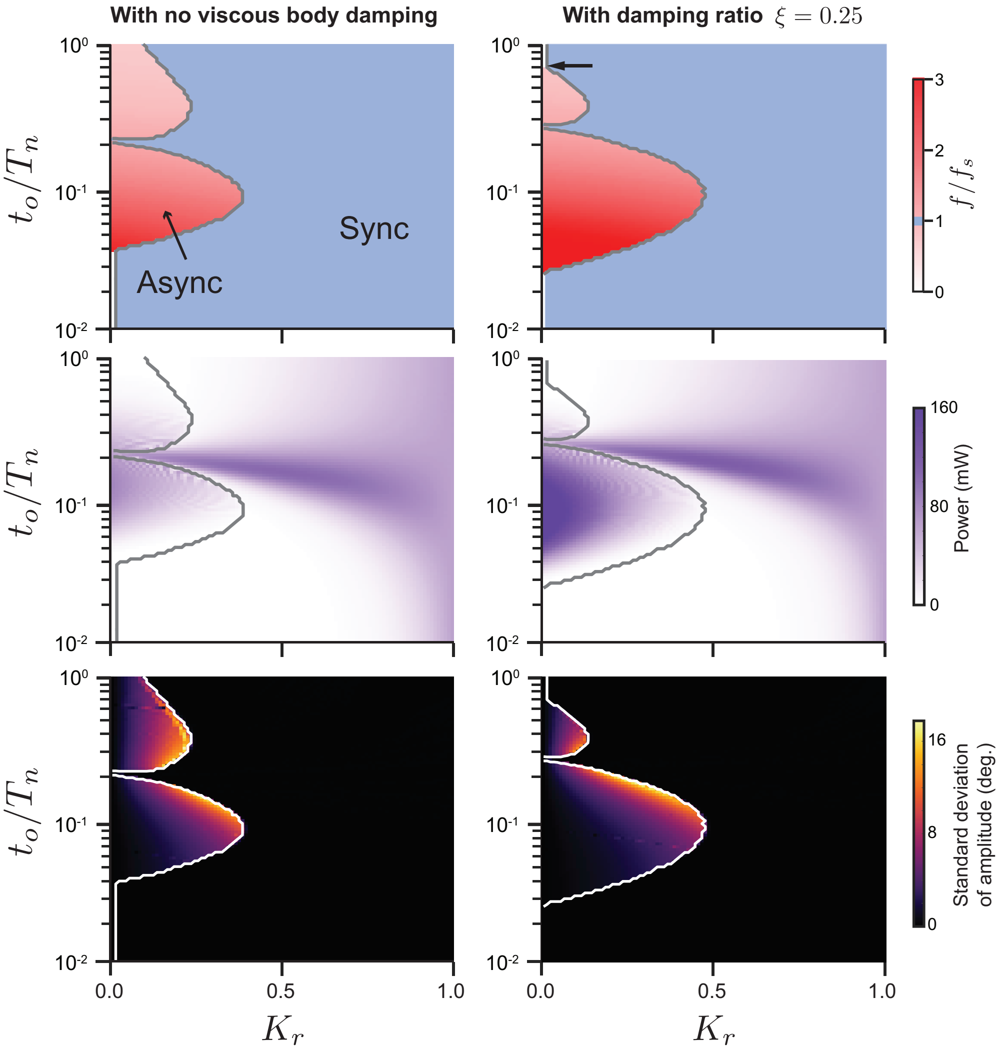

FIG. SI5. **Comparison of simulation results with and without body viscous damping.** Top row shows emergent frequency, middle row shows power, and bottom row shows the standard deviation of peak-to-peak amplitude. Gray and white lines designate boundaries between asynchronous and synchronous modes. Both simulations display the same fundamental phenomena: 1) asynchronous oscillations emerge as the normalized time-to-peak of the delayed stretch activation response (t_0/T_n) increases, and 2) a bridge of smooth sinusoidal wingbeats exists between the asynchronous and synchronous modes of actuation. However, viscous body damping results in the suppression of emergent asynchronous wingbeats for larger t_0/T_n as indicated by the arrow in the upper right plot.

In Fig. SI5 we compare here simulation results for $\xi = 0$ (as in the main text) and a modest damping of $\xi = 0.25$. With viscous damping, we observe the same fundamental phenomena, the presence of a bridge in parameter space between the asynchronous and synchronous regimes. However, in the viscously damped case, we do observe an upper limit for t_0/T_n where emergent oscillations disappear (the same phenomena as observed in robophysical experiment). Thus, the presence of viscous damping can influence the generation of asynchronous wingbeats and as the time to reach peak force for delayed stretch activation increases, viscous dissipation can suppress asynchronous wingbeats.

[1] K. J. Tong, S. Duchêne, S. Y. W. Ho, and N. Lo, Comment on "phylogenomics resolves the timing and pattern of insect evolution", Science **349**, 487 (2015).
[2] B. Misof, R. G. Beutel, O. Niehuis, M. Petersen, F. Izquierdo-Carrasco, T. Wappler, J. Rust, S. Liu, K. Meusemann, R. S. Peters, A. Donath, C. Mayer, P. B. Frandsen, J. Ware, and T. Flouri, Phylogenomics resolves the timing and pattern of insect evolution, Science **346**, 763 (2014).
[3] L. J. Revell, phytools: an r package for phylogenetic comparative biology (and other things), Methods in Ecology and Evolution **3**, 217 (2012).
[4] J. M. Beaulieu, B. C. O'Meara, and M. J. Donoghue, Identifying hidden rate changes in the evolution of a binary morphological character: The evolution of plant habit in campanulid angiosperms, Systematic Biology **62**, 725 (2013).
[5] J. D. Boyko and J. M. Beaulieu, Generalized hidden Markov models for phylogenetic comparative datasets, Methods in Ecology and Evolution **12**, 468 (2021).
[6] B. King and M. S. Lee, Ancestral state reconstruction, rate heterogeneity, and the evolution of reptile viviparity, Systematic Biology **64**, 532 (2015).
[7] R. Dudley, *The Biomechanics of Insect Flight. Form, Function, Evolution* (Princeton University Press, 2000) p. 476.
[8] R. K. Josephson, Mechanical power output from striated-muscle during cyclic contraction, Journal of Experimental Biology **114**, 493 (1985).
[9] A. N. Ahn, How Muscles Function - the Work Loop Technique, Journal of Experimental Biology **215**, 1051 (2012).
[10] R. K. Josephson, J. G. Malamud, and D. R. Stokes, Power output by an asynchronous flight muscle from a beetle., Journal of Experimental Biology **203**, 2667 (2000).
[11] J. Molloy, V. Kyrtatas, J. C. Sparrow, and D. C. S. White, Kinetics of flight muscles from insects with different wingbeat frequencies, Nature **328**, 449 (1987).
[12] J. Gau, N. Gravish, and S. Sponberg, Indirect actuation reduces flight power requirements in Manduca sexta via elastic energy exchange, Journal of the Royal Society Interface **16**, 20190543 (2019).
[13] N. T. George, T. C. Irving, C. D. Williams, and T. L. Daniel, The Cross-Bridge Spring: Can Cool Muscles Store Elastic Energy?, Science **340**, 1217 (2013).
[14] J. Gau, E. S. Wold, J. Lynch, N. Gravish, and S. Sponberg, enThe hawkmoth wingbeat is not at resonance, Biology Letters **18**, 20220063 (2022).
[15] M. S. Tu and T. L. Daniel, Submaximal power output from the dorsolongitudinal flight muscles of the hawkmoth Manduca sexta, Journal of Experimental Biology **207**, 4651 (2004).
