# Supplementary material for: Bridging two insect flight modes in evolution, physiology and robophysics
Source: Nature. 2023 Oct 4;622(7984):767–74. doi: 10.1038/s41586-023-06606-3 (PMC10599994; doi:10.1038/s41586-023-06606-3)
Supplement: Supplementary file 2 — Reporting Summary [file 41586_2023_6606_MOESM2_ESM.pdf]

nature portfolio

Corresponding author(s): Simon Sponberg & Nick Gravish

Last updated by author(s): Jul 9, 2023

Reporting Summary

Nature Portfolio wishes to improve the reproducibility of the work that we publish. This form provides structure for consistency and transparency in reporting. For further information on Nature Portfolio policies, see our Editorial Policies and the Editorial Policy Checklist.

Statistics

For all statistical analyses, confirm that the following items are present in the figure legend, table legend, main text, or Methods section.

n/a	Confirmed	
☐	☒	The exact sample size (*n*) for each experimental group/condition, given as a discrete number and unit of measurement
☐	☒	A statement on whether measurements were taken from distinct samples or whether the same sample was measured repeatedly
☒	☐	The statistical test(s) used AND whether they are one- or two-sided<br>*Only common tests should be described solely by name; describe more complex techniques in the Methods section.*
☒	☐	A description of all covariates tested
☒	☐	A description of any assumptions or corrections, such as tests of normality and adjustment for multiple comparisons
☐	☒	A full description of the statistical parameters including central tendency (e.g. means) or other basic estimates (e.g. regression coefficient) AND variation (e.g. standard deviation) or associated estimates of uncertainty (e.g. confidence intervals)
☒	☐	For null hypothesis testing, the test statistic (e.g. *F*, *t*, *r*) with confidence intervals, effect sizes, degrees of freedom and *P* value noted<br>*Give P values as exact values whenever suitable.*
☒	☐	For Bayesian analysis, information on the choice of priors and Markov chain Monte Carlo settings
☒	☐	For hierarchical and complex designs, identification of the appropriate level for tests and full reporting of outcomes
☒	☐	Estimates of effect sizes (e.g. Cohen's *d*, Pearson's *r*), indicating how they were calculated

Our web collection on statistics for biologists contains articles on many of the points above.

Software and code

Policy information about availability of computer code

Data collection	Matlab and Simulink (R2020, Mathworks) was used for all data collection as well as robophysical and robot platforms.
Data analysis	All phylogenetic analyses were done using R studio (v. 1.1.383) using R (v. 4.0.2), with the PhyTools and corHMM packages. No special data analysis was required for the physiology data. Plotting was done with Matlab (R2020). All code for implementing the simulations, robophyiscal model and robotic platform are available in the github repository: https://github.com/agilesystemslab/synch_asynch_sim

For manuscripts utilizing custom algorithms or software that are central to the research but not yet described in published literature, software must be made available to editors and reviewers. We strongly encourage code deposition in a community repository (e.g. GitHub). See the Nature Portfolio guidelines for submitting code & software for further information.

Data

Policy information about availability of data

All manuscripts must include a data availability statement. This statement should provide the following information, where applicable:

- Accession codes, unique identifiers, or web links for publicly available datasets
- A description of any restrictions on data availability
- For clinical datasets or third party data, please ensure that the statement adheres to our policy

Muscle ultrastructure data that was collected from prior literature is located in supplementary data table 1. Raw physiological data for the muscle experiments are available at the Georgia Tech SmartTech data repository: http://hdl.handle.net/1853/66777

Research involving human participants, their data, or biological material

Policy information about studies with human participants or human data. See also policy information about sex, gender (identity/presentation), and sexual orientation and race, ethnicity and racism.

Reporting on sex and gender	N/A
Reporting on race, ethnicity, or other socially relevant groupings	N/A
Population characteristics	N/A
Recruitment	N/A
Ethics oversight	N/A

Note that full information on the approval of the study protocol must also be provided in the manuscript.

Field-specific reporting

Please select the one below that is the best fit for your research. If you are not sure, read the appropriate sections before making your selection.

☒ Life sciences ☐ Behavioural & social sciences ☐ Ecological, evolutionary & environmental sciences

For a reference copy of the document with all sections, see nature.com/documents/nr-reporting-summary-flat.pdf

Life sciences study design

All studies must disclose on these points even when the disclosure is negative.

Sample size	Sample size of 9 independent individuals from the same source colonies were chosen based on prior muscle physiological experiments such as: Tu & Daniel 2004, Journal of Experimental Biology; Wang, et al. 2018, Biophysical Journal; Josephson, 1997, Journal of Experimental Biology
Data exclusions	No data were excluded from the muscle physiology experiments.
Replication	All experimental protocols were identical and replicated across each of 9 preparations. No further replication was conducted because other colony sources were not available and results were consistent across the individuals analyzed.
Randomization	Twitch responses were characterized first in all preparations to determine that stimulation was effective. All other measurements were taken in a continuous ramp & hold trial, so there was no need for randomization.
Blinding	The experiments were not blinded because there were not multiple group allocations of data being collected. The researcher also had to monitor the data during experiment to determine if stimulation of the muscle produced a force response.

Reporting for specific materials, systems and methods

We require information from authors about some types of materials, experimental systems and methods used in many studies. Here, indicate whether each material, system or method listed is relevant to your study. If you are not sure if a list item applies to your research, read the appropriate section before selecting a response.

Materials & experimental systems

n/a	Involved in the study	
☒	☐	Antibodies
☒	☐	Eukaryotic cell lines
☒	☐	Palaeontology and archaeology
☐	☒	Animals and other organisms
☒	☐	Clinical data
☒	☐	Dual use research of concern
☒	☐	Plants

Methods

n/a	Involved in the study	
☒	☐	ChIP-seq
☒	☐	Flow cytometry
☒	☐	MRI-based neuroimaging

Animals and other research organisms

Policy information about studies involving animals; ARRIVE guidelines recommended for reporting animal research, and Sex and Gender in Research

Laboratory animals	Adult Manduca sexta (2-6 days post eclosion) from two colonies (University of Washington and Case Western Reserve Univ.)
Wild animals	No wild animals used
Reporting on sex	Both sexes were used in this study (6 females and 3 males).
Field-collected samples	No Field collected samples were used.
Ethics oversight	All animals used were invertebrates and not governed by IACUC guidelines.

Note that full information on the approval of the study protocol must also be provided in the manuscript.
