# Supplementary material for: Bridging two insect flight modes in evolution, physiology and robophysics
Source: Nature. 2023 Oct 4;622(7984):767–74. doi: 10.1038/s41586-023-06606-3 (PMC10599994; doi:10.1038/s41586-023-06606-3)
Supplement: Supplementary file 3 — Hawkmoth simulation. Definitions and values of variables used in simulation. [file 41586_2023_6606_MOESM3_ESM.docx]

| variable | value | description |
| --- | --- | --- |
| *k* | 2582 ± 510 N m*^−^*^1^ | thoracic stiffness[^39^](#_1v1yuxt) |
| *I* | 5.69± 0.34 E-8 kg m^2^ rad*^−^*^1^ | wing inertia |
| *T* | 2230 ± 110 rad m*^−^*^1^ | transmission ratio |
| Γ | 3.69 ± 0.33 E-8 kg m^2^ rad*^−^*^2^ | damping coefficient |
| *R*_2_(*m*) | 0.383 ± 0.003 | 2nd moment of wing area[^24^](#_qsh70q) |
| *R*_2_(*v*) | 0.482 ± 0.001 | 2nd moment of added mass[^24^](#_qsh70q) |
| *R*_2_(*s*) | 0.518 ± 0.001 | 2nd moment of wing shape[^24^](#_qsh70q) |
| *m_w_* | 0.092 g | wing mass (both wings)[^24^](#_qsh70q) |
| *v*ˆ | 1.08 | added mass[^24^](#_qsh70q) |
| *ρ* | 1.225 kg m*^−^*^3^ | air density |
| *A_w_* | 1881 mm^2^ | wing area (both)[^24^](#_qsh70q) |
| (AR) | 5.53 ± 0.04 | wing aspect ratio (both wings)[^24^](#_qsh70q) |
| *L_w_* | 51 mm | wing length[^24^](#_qsh70q) |
| *_C_*˜*_D_* | 1.5 | drag coefficient[^75^](#_48pi1tg) |
| *lcp* | 30.6 mm | aerodynamic center of pressure[^75^](#_48pi1tg) |
| *ϕ*_0_ | 117 ± 6° | peak-to-peak wingstroke amp[^24^](#_qsh70q) |
| *X*_0_ | 0.46 ± 0.02 mm | peak-to-peak muscle displacement amp[^18^](#_1y810tw) |
| *F_s_* | 2.72 N | zero-to-peak muscle force amp |
