# Supplementary material for: Bridging two insect flight modes in evolution, physiology and robophysics
Source: Nature. 2023 Oct 4;622(7984):767–74. doi: 10.1038/s41586-023-06606-3 (PMC10599994; doi:10.1038/s41586-023-06606-3)
Supplement: Supplementary file 4 — Robophysics experiment. Dynamically scaled robophysical system parameters. [file 41586_2023_6606_MOESM4_ESM.docx]

| variable | value | description |
| --- | --- | --- |
| *k* | 0.525 N m | stiffness |
| *I* | 0.0023 kg m^2^ | wing+system inertia |
| Γ | 0.0017 kg m^2^ | damping torque coefficient |
| *T* | 1 | transmission ratio |
