# Supplementary material for: Bridging two insect flight modes in evolution, physiology and robophysics
Source: Nature. 2023 Oct 4;622(7984):767–74. doi: 10.1038/s41586-023-06606-3 (PMC10599994; doi:10.1038/s41586-023-06606-3)
Supplement: Supplementary file 5 — Robobee parameters. Parameters used in robobee experiment. [file 41586_2023_6606_MOESM5_ESM.docx]

| variable | value | Description |
| --- | --- | --- |
| *k* | 197.7 N m | Stiffness |
| *I* | 5.55E-11 kg m^2^ | wing inertia |
| Γ | 5.68E-11 kg m^2^ | damping coefficient |
| *T* | 3333 rad m*^−^*^1^ | transmission ratio |
